# Sparing sentinel node biopsy through axillary lymph node fine needle aspiration in primary breast cancers

**DOI:** 10.1186/1477-7819-11-296

**Published:** 2013-11-20

**Authors:** Yu-Shu Cheng, Shou-Jen Kuo, Dar-Ren Chen

**Affiliations:** 1 Department of Surgery, Changhua Christian Hospital, 135 Nanhsiao Street, Changhua 50006, Taiwan; 2 Comprehensive Breast Cancer Center, Changhua Christian Hospital, Changhua 50006, Taiwan; 3 School of Medicine, Chung Shan Medical University, Taichung 40201, Taiwan

**Keywords:** Axillary lymph node, Breast cancer, Fine needle aspiration cytology

## Abstract

**Background:**

Axillary lymph node status is an important staging and prognostic factor in breast cancer. This study aimed to evaluate the efficacy of axilla fine needle aspiration cytology (FNAC) in primary breast cancer without a palpable node and even without image characteristics of a metastatic node.

**Methods:**

From June 2008 to January 2012, 77 patients met the inclusion criteria of having received a FNAC procedure during the diagnostic protocol of primary breast cancer with the characteristic of impalpable axilla nodes, and of having received axillary surgery after that, according to the guidelines. The patients’ characteristics, clinical-pathological features, pre-operative axillary lymph node FNAC findings, surgical lymph node report, and definite pathologic staging were reviewed.

**Results:**

The FNAC procedures had a reported sensitivity of 58.82%, specificity of 100%, positive predictive value of 100%, negative predictive value of 72.55%, and accuracy of 80.28%. There were no false positives on FNAC; therefore, the positive likelihood ratio approached infinity. The negative likelihood ratio was 41.18%. Axillary lymph node FNAC is feasible in newly diagnosed breast cancer patients to evaluate metastatic lymph nodes even in those without clinical or ultrasonic evidence of lymphadenopathy.

**Conclusions:**

FNAC can be a routine evaluation for most primary breast cancer patients with benefits in expediting treatment. For those patients with positive findings of the axilla, sentinel node biopsy can be avoided.

## Background

Axillary lymph node status in the initial staging of newly diagnosed breast carcinoma is important for prognosis and assessment of treatment options. Though specific cortical and hilar echo-morphologic changes have been shown to be predictive of metastatic lymph nodes [1-3], tissue proof is the main guide to providing definite staging. Axillary lymph node dissection (ALND) has been a standard part of breast cancer treatment, but concerns have arisen following complications such as lymphedema, decreased range of motion in the shoulder, and paresthesia. Therefore, sentinel lymph node biopsy (SLNB) was developed to decrease morbidity and avoid unnecessary ALND [4,5]. Nevertheless, SLNB is still an invasive, technique-dependent, and time-consuming procedure.

Fine needle aspiration cytology (FNAC) of suspicious nodes via clinical examination and/or ultrasound has become a popular practice in many breast units. It is performed without anesthesia and is generally well tolerated by the patient, with fewer complications than needle core biopsy. In most of the reported series, the focus has been on patients with a suspicious axillary node identified by ultrasonography or who had palpable lymph nodes and then underwent FNAC. This selection may reduce the bias of the operator-dependent procedure, but limits the application with regards to primary breast cancer patients.

In the present study, we performed the procedure regardless of clinical or ultrasonic characteristics. FNAC was applied routinely even for those without clinical or imaging evidence of node metastasis in a highly suspicious breast malignancy. Cytology results were compared with pathology after SLNB or ALND.

The aim of the study was to determine whether axillary lymph node ultrasound-guided FNAC can be a safe, feasible, time-saving, sensitive, and specific tool to predict metastatic lymph nodes in primary breast cancer without palpable axillary lymph nodes or even imaging characteristics of a metastatic node.

## Methods

Since June 2008, axillary lymph node ultrasound and FNAC have been used routinely along with core needle biopsy by an experienced breast surgeon (more than 20 years’ experience in breast ultrasound examination) for all patients highly suspicious of having a malignant lesion of the breast to evaluate the lymph node metastasis status.

All patients with or without a palpable axillary lymph node received an axilla echogram followed by FNAC. The aspirational target was the node over the low axilla (level I), which is the usual location of the sentinel lymph node. Sonographic characteristics may be taken as a reference, but are not essential. We aspirated the node with evacuation using 22 gauge needles. The specimen was fixed to the slide with 95% alcohol and was reviewed by a pathologist. A positive finding was defined as the presentation of malignant cells. Regardless of the aspiration cytology results, the patients underwent axillary surgery according to the guidelines for the definite histopathology report.

All patients who underwent the technique between June 2008 and January 2012 and gave their consent were enrolled into the specific registry database. Of the 161 patients who received axillary lymph node FNAC during this time, 84 met the exclusion criteria (3 patients underwent breast tumor core needle biopsy at the same time, revealing a benign lesion; 16 had non-primary breast cancer; 24 did not undergo surgical intervention; and 41 received neo-adjuvant therapy before the operation) and were excluded; 77 patients were finally included in the study.

This study focuses on the group of patients diagnosed with primary breast cancer followed by lymph node operation; whether SLNB or ALND were chosen depended on the surgeon’s preference. The exclusion criteria included a benign lesion in the biopsy report, non-primary breast cancer, patients without an operation, and patients who received neoadjuvant therapy before the operation. The entire protocol was approved by the Institutional Review Board of the hospital.

The patient’s characteristics, clinicopathological features, pre-operative axillary lymph node FNAC findings, surgical lymph node report, and definite pathologic staging were reviewed. To evaluate the efficacy of this diagnostic tool, sensitivity, specificity, accuracy, positive predictive value (PPV), and negative predictive value (NPV) were calculated.

## Results

### Patient characteristics

All 77 patients were female with a mean age of 53.09 years, ranging from 27 to 76 years. A unilateral lesion was noted in the patients, 33 (42.86%) on the right side and 44 (57.14%) on the left. Three (3.9%) patients had lymph nodes suspicious of metastasis in sonograms. Most patients presented with an expression of hormone receptor (52 of 77 patients had ER+, 52 had PR+), but less HER2 gene amplification (25 of 77, 32.47%) (Table 1).

**Table 1:** Patient demographic data

**Characteristic**	**Total (n = 77)**	**%**
Age, yr		
Mean (SD)	53.09 (11.31)	
Range	27.16–75.72	
Tumor size, cm		
Mean (SD)	2.44 (1.34)	
Range	0.10–7.00	
Tumor side		
Right	33	42.86
Left	44	57.14
Tumor histological type		
Ductal	72	93.51
Lobular	4	5.19
Other	1	1.3
Tumor grading		
1	7	9.09
2	41	53.25
3	25	32.47
Not applicable	4	5.19
ER		
Positive	52	67.53
Negative	25	32.47
PR		
Positive	52	67.53
Negative	25	32.47
Her2		
Positive	25	32.47
Negative	52	67.53
Cytology report		
Malignancy	20	25.97
Negative for malignant cells	51	66.23
Atypia	4	5.19
Unsatisfactory specimen	2	2.6
Surgical pathology of LN		
Positive	38	49.35
Negative	39	50.65
Tumor size (pT staging)		
Tis	4	5.19
T1mi	3	3.9
T1a	3	3.9
T1b	2	2.6
T1c	20	25.97
T2	38	49.35
T3	6	7.79
T4	1	1.3
Lymph node number (pN staging)		
N0	39	50.65
N1	24	31.17
N2	10	12.99
N3	4	5.19
Lymphovascular invasion		
Present	46	59.74
Not identified	20	25.97
Not available	11	14.29

### Tumor histopathologic characteristics

Gross tumor size was recorded in the pathology report of the surgical specimen, with sizes ranging from 10 to 70 mm (mean size, 24.4 mm). Most tumors were staged as T2 (38 of 77 patients, 49.35%) and T1 (28 of 77, 36.36%) lesions. There were 4 Tis, 6 T3, and 1 T4 lesion in the pathologic T staging statistics. Of the primary breast carcinoma types, 72 (93.51%) were ductal, 4 (5.19%) were lobular, and 1 (1.3%) was reported to be a papillary lesion with ductal carcinoma in situ. In the tumor grading system, 53.25% patients were grade 2 and 32.47% were grade 3 (Table 1).

### Axillary lymph node FNAC findings

The included patients all had primary breast cancer and had undergone axillary lymph node aspiration cytology at the same time or right after tumor biopsy. A “positive” result was defined as the presence of malignant cells (20 of 77 patients, 25.97%) and a “negative” result as no malignant cells (51 of 77 patients, 66.23%). Six patients presented with “borderline” findings inclusive of atypia and an unsatisfactory specimen that could not be categorized as positive or negative (Table 2), so those patients were excluded when calculating the efficacy of the diagnostic tool.

**Table 2:** Results of fine needle aspiration cytology of axillary lymph nodes

	**Fine needle aspiration cytology**		
**Axillary procedure**	**Positive**	**Negative**	**Borderline**
SLNB	3	46	4
Lymph node-positive	3	13	3
ALND	17	5	1
Lymph node-positive	17	1	1
NA	–	–	1

SLNB: Sentinel lymph node biopsy; ALND: Axillary lymph node dissection.

### Lymph node management and characteristics

All patients underwent axillary surgery for the definite histopathologic report. The method of operation was determined by the surgeon’s experience and preference. As shown in Table 2, 3 of the 20 patients with a positive FNAC received SLNB and 17 received ALND. All of the pathologic reports showed evidence of lymph node metastasis. Of the 51 patients with a negative FNAC result, 46 received SLNB, and 13 had lymph node metastasis. One of the 5 patients receiving ALND had lymph node involvement.

### Diagnostic tool efficacy

The FNAC procedures with 20 positive and 51 negative results were compared with the surgical pathology reports, both SLND and ALND, to evaluate the efficacy of the diagnostic tool. There were 34 positive and 37 negative lymph nodes in the surgical pathologic reports. In the statistical analysis, sensitivity was 58.82%, specificity 100%, PPV 100%, and NPV 72.55%; the false-positive rate (FPR) was 0%, and the false-negative rate (FNR) was 41.18% (Table 3).

**Table 3:** Efficacy of the diagnostic tools

	**Lymph node in surgical pathology (SLNB or ALND)**	
	**Positive**	**Negative**
FNAC positive	20	0
FNAC negative	14	37
Sensitivity	58.82%	
Specificity	100%	
Positive predictive value	100%	
Negative predictive value	72.55%	
False-positive rate	0%	
False-negative rate	41.18%	
Accuracy	80.28%	

SLNB: Sentinel lymph node biopsy; ALND: Axillary lymph node dissection; FNAC: Fine needle aspiration cytology.

## Discussion

This study demonstrates the benefits of performing axillary lymph node FNAC, especially in the routine examination of every primary breast cancer patient. We focused on the efficacy index of this diagnostic tool to determine if it improves the detection of metastatic disease and alters the decision-making for the patient’s treatment.

Lymph node metastasis status is an important factor in the prognosis of breast cancer. There are various ways to determine it. To evaluate the efficacy of a diagnostic technique, sensitivity, specificity, accuracy, PPV, and NPV are the important indicators. Physical examination via axillary lymph node palpation is the basic tool to evaluate the metastatic lymph node, but it has a low specificity and low sensitivity of 32% to 68%, with a high false negative rate of 45% [6-10]. Due to the suboptimal subjective judgment of clinical palpation, imaging assessment was investigated to provide objective evidence. Computed tomography, magnetic resonance imaging, and nuclear medicine techniques can visualize the axillary lymph nodes, but ultrasound is the most advantageous technique and is the most commonly used to characterize lymph nodes and detect axillary lymph node metastases [3,6,7,11]. This broadly favored technique offers a wide variation in sensitivity, with a range of 30% to 92%, and specificity in the range of 69% to 100% [8,9,12]. Some criteria, including size, morphology, color, and pattern, are used to assess a lymph node via ultrasound [13]. However, it is operator-dependent and lacks technique standardization. The axillary lymph node pathologic report provides objective and reliable node metastasis information. ALND or axillary node clearance has traditionally been a routine management for pathologic examination and radical treatment. However, the anatomic disruption caused by ALND may result in infection, hematoma, seroma, arm morbidity, and nerve injury, which compromise functionality and quality of life. The risk is significantly less for SLNB [14], and it has become an alternative method for patients who have clinically negative axillary lymph nodes, thereby obviating the need for more extensive surgery. The FNR of SLNB is generally accepted to lie between 5% and 10% [15]. FNAC has been developed and performed in many breast units. The procedure is less invasive than SLNB and has fewer complications, and is applied mostly on patients with suspicious nodes via clinical examination and/or ultrasound.

This study enrolled 77 patients who had undergone FNAC procedures performed routinely in cases of primary breast cancer with clinically negative axillary lymph nodes. FNAC was performed regardless of ultrasound characteristics. Our results demonstrated a sensitivity of 58.82%, specificity of 100%, PPV of 100%, NPV of 72.55%, and an accuracy of 80.28%. There were no false positives on FNAC; therefore, the positive likelihood ratio approached infinity. It should be noted that FNAC, as a diagnostic tool, provides very high specificity resulting in the excellent predictive power of a positive result.

A number of prospective studies looking at the role of ultrasound-guided sampling of axillary lymph nodes in breast cancer have been published [8,16-18]. Reported sensitivities and specificities in the literature were 40% to 87%, and 56% to 100%, respectively [16]. In the previous study by Marti et al. [17], the axillary FNAC was 86% sensitive, 100% specific, and 91% accurate; PPV was 100% and NPV was 78%. Rattay et al. [16] found that in FNAC of suspicious nodes to determine nodal involvement, sensitivity was 76%, specificity was 100%, PPV was 100%, and NPV was 48%. Chang et al. [18] reported a positive lymph node FNAC had a PPV of 98.7%. Hayes et al. [8] illustrated that FNAC had a sensitivity for axillary involvement of 66.3%, and a specificity, PPV, and NPV of 98.7%, 98.3%, and 71.8%, respectively. In contrast, our results manifested lower sensitivity (58.82%), identical specificity (100%), and higher PPV (100%) and NPV (72.55%); moreover, there was an extremely low FPR (0%) and a low FNR (41.18%).

The main advantage of axillary lymph node FNAC is the positive result. Once evidence of a malignant cell is found in a patient who receives the examination, treatment can be expedited. In sparing the SLNB procedure, the patient proceeds directly to ALND or neoadjuvant chemotherapy. The advantages include not having to wait for the definitive pathologic response of the sentinel node and, for those indicated for neoadjuvant chemotherapy, avoiding a second surgery. Low sensitivity and negative results would not alter the current treatment guidelines and protocols.

There are limitations and possible further developmental directions in our study. FNAC via ultrasound is operator-dependent to such an extent that sampling may vary. A unified protocol should be established. In addition, the enrolled number of patients was relatively small, leading to difficulty in evaluating the atypia group. To combine other diagnostic tools with the design criteria may interfere with sensitivity. In addition, cost data should be collected to evaluate cost-effectiveness. Furthermore, comparative studies and meta-analyses may be required to identify adequate sensitivity, accuracy, and FNR in order to make the tool worthwhile.

## Conclusions

Axillary lymph node FNAC is a tool with rapid access and is an office-based device that can be an attractive alternative in the evaluation of lymph node involvement in newly diagnosed breast cancer patients. It may be applied routinely before operation due to its high specificity and PPV, and may play a role in sparing SNLB and proceeding to ALND or neoadjuvant chemotherapy.

## Abbreviations

ALND: Axillary lymph node dissection; FNAC: Fine needle aspiration cytology; FNR: False-negative rate; FPR: False-positive rate; NPV: Negative predictive value; PPV: Positive predictive value; SLNB: Sentinel lymph node biopsy.

## Competing interests

The authors declare that they have no competing interests.

## Authors’ contributions

D-RC conceived of the study, participated in its design, and helped to draft the manuscript. Y-SC participated in the design of the study, performed the statistical analysis, and drafted the manuscript. S-JK helped to revise the manuscript. All authors read and approved the final manuscript.
